# Chromatin Structure Following UV-Induced DNA Damage—Repair or Death?

**DOI:** 10.3390/ijms12118063

**Published:** 2011-11-17

**Authors:** Andrew W. Farrell, Gary M. Halliday, James Guy Lyons

**Affiliations:** Discipline of Dermatology, Bosch Institute, Sydney Cancer Centre, The University of Sydney, NSW 2006, Australia; E-Mails: andrew.farrell@sydney.edu.au (A.W.F.); guy.lyons@sydney.edu.au (J.G.L.)

**Keywords:** chromatin, UV, apoptosis, repair, histone, SWI/SNF

## Abstract

In eukaryotes, DNA is compacted into a complex structure known as chromatin. The unravelling of DNA is a crucial step in DNA repair, replication, transcription and recombination as this allows access to DNA for these processes. Failure to package DNA into the nucleosome, the individual unit of chromatin, can lead to genomic instability, driving a cell into apoptosis, senescence, or cellular proliferation. Ultraviolet (UV) radiation damage causes destabilisation of chromatin integrity. UV irradiation induces DNA damage such as photolesions and subjects the chromatin to substantial rearrangements, causing the arrest of transcription forks and cell cycle arrest. Highly conserved processes known as nucleotide and base excision repair (NER and BER) then begin to repair these lesions. However, if DNA repair fails, the cell may be forced into apoptosis. The modification of various histones as well as nucleosome remodelling via ATP-dependent chromatin remodelling complexes are required not only to repair these UV-induced DNA lesions, but also for apoptosis signalling. Histone modifications and nucleosome remodelling in response to UV also lead to the recruitment of various repair and pro-apoptotic proteins. Thus, the way in which a cell responds to UV irradiation via these modifications is important in determining its fate. Failure of these DNA damage response steps can lead to cellular proliferation and oncogenic development, causing skin cancer, hence these chromatin changes are critical for a proper response to UV-induced injury.

## 1. Chromatin

Eukaryotic cells compact their DNA into repeating arrays of nucleosomes comprised of 146 bp of DNA wrapped around an octamer of histone proteins—a tetramer of H3 and H4 flanked by two H2A and H2B dimers [1], otherwise known as chromatin. A fifth histone protein, H1, promotes chromatin structure of a higher order by facilitating the compaction of nearby nucleosomes at the entry and exit points. “Linker DNA” connects these core proteins, which is typically 30–50 bp in length [2]. Chromatin has been subdivided into two functionally distinct classes. Firstly, euchromatin which is decondensed in the interphase of the cell cycle and contains most of the actively transcribed genes, and secondly heterochromatin which remains condensed throughout the whole cell cycle and contains mostly inactive genes [3,4]. Each core histone contains a globular domain and an *N*-terminal tail protruding from the nucleosome. The nucleosome is in a constant dynamic equilibrium between a fully wrapped state and a collection of partially unwrapped states. This unwrapping transiently exposes buried DNA sites for access. However the conversion between these states is dynamic and rapid. Any process that requires access to DNA must therefore overcome this chromatin structural barrier [5].

This nucleosomal structure is the platform for which many variations, modifications and binding of proteins all impact DNA and cell functions. A number of chromatin remodelling complexes use adenosine 5′-triphosphate (ATP) to alter chromatin structure, making the DNA accessible to the proteins which bind chromatin and regulate cellular processes. This can influence the epigenetic modifications of various histones, as well as the alteration of the *N*-terminal tail of chromatin by histone chaperones, in turn influencing the dynamic state of chromatin [6]. These chromatin-remodelling factors are important in the link between chromatin structure and cellular processes such as transcription, replication, recombination, aging, repair, cell cycle control, death signalling and responses to external stimuli such as ultraviolet (UV) radiation.

## 2. UV Damage and the Cellular Response

Chromatin is under constant threat from both endogenous and exogenous sources. Among these sources, UV radiation is one of the most prevalent inducers of DNA damage in our environment. The photolesions induced by UV inhibit the replication of DNA, as well as transcription thus causing genomic instability [7]. If they are not repaired prior to cell division, these photolesions can result in an incorrect nucleotide, or mutation, being incorporated into newly synthesised DNA. When UV reaches the skin, a major part of it is reflected, while the rest is absorbed. UVB radiation (290–320 nm) is absorbed mostly by the upper epidermal layers, while UVA (320–400 nm) penetrates deeper into the skin, reaching the basal layer of the epidermis [8]. UVB radiation has been shown to cause different types of DNA damage, including the formation of dimeric photoproducts between adjacent pyrimidine dimers on each strand. There are two major types of these lesions induced by UV irradiation: cyclobutane pyrimidine dimers (CPDs) and Pyrimidine (6–4) Pyrimidone photoproducts (6-4PPs). Although UVA has also been shown to result in the formation of pyrimidine dimers [9,10], it requires much more energy than UVB to incur this damage. However, UVA causes greater levels of oxidative damage than UVB, and has been shown to cause increased 8-oxo-deoxyguanine (8-oxo-dG) lesions, which result from the oxidation of deoxyguanine moieties [11].

### 2.1. DNA Repair

To counteract these DNA lesions, eukaryotes have evolved a number of processes that make it possible for the cell to recover from damage. The inherent biological tendency is to maintain genetic homeostasis. To accomplish this, DNA damage induces cellular responses leading to cell cycle arrest. A cell can repair or tolerate the DNA damage, or the cells that harbor damage can be removed from the population via induced death. Diverse DNA repair pathways have been conserved in a large number of species as they have evolved over time to repair different forms of lesions [3]. The nucleotide excision repair (NER) pathway is primarily involved in removing helix-distorting lesions such as CPDs and 6-4PPs induced by UV [12]. Upon UV radiation, cells are arrested in the G1 and G2 phases of the cell cycle, both of which are regulated by the tumour suppressor P53 [13]. In eukaryotic cells, the NER pathway is subdivided into two pathways: global genome repair (GGR), which repairs lesions located anywhere within the genome, and transcription coupled repair (TCR) which repairs genes in the transcribed strand of active genes [14]. In mammals, congenital defects in GGR and TCR lead to an increased sensitivity towards DNA damaging agents such as UV and disorders such as Cockayne’s Syndrome [15]. Both pathways have a core set of repair factors involved, but have specific factors involved in the initial stages of recognition. GGR is initiated by the activation of several DNA-damage binding proteins, most importantly the XPC-HR23B centrin-2 complex and/or the DDB1-DDB2 (DNA damage binding protein 1 and 2) heterodimer complex [16]. Repair of damage in the transcribed strands (TCR) is initiated by two distinct damage recognition mechanisms. Both the ATP-dependent chromatin remodelling protein Cockayne Syndrome protein B (CSB) and the WD40 domain containing protein Cockayne Syndrome protein A (CSA) are recruited to the site of damage [17]. TCR is then theorised to be initiated by the abrogation of RNA polymerase II during transcription [18].

**Figure 1:**
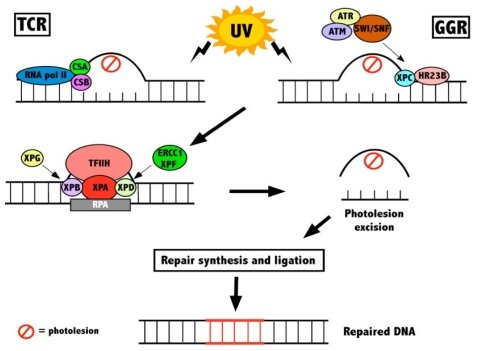
The mechanisms of NER following UV radiation. Photolesions are recognised by both TCR and GGR, both of which have specific recognition factors involved. These two pathways then integrate, with several repair proteins assembling at the site of damage, unwinding the DNA, and orienting the excision of the photolesion. This is then followed by new DNA synthesis and ligation.

Subsequent steps are performed by a common set of NER factors that are shared by GGR and TCR. This involves the unwinding of the DNA duplex at the sites of damage by Xeroderma Pigmentosum group B and D helicases (XPB and XPD), which are subunits of the transcription factor IIH (TFIIH) complex [19,20]. XPA is then recruited to stabilise the repair complex and to orient the dual incision of the DNA lesion by two structure-specific endonucleases, XPG and ERCC1-XPF, leading to the removal of a section of single stranded DNA with a gap of 25–30 nucleotides. This gap is then filled by DNA synthesis and ligation via DNA polymerase δ/ε, replication factor C (RFC), PCNA, RPA and DNA ligase I (Figure 1) [21,22].

Another form of repair named base excision repair (BER) is responsible for removing small, single base non-helix-distorting oxidative lesions from DNA, commonly induced by UVA radiation [23]. These oxidative lesions are induced by highly reactive oxygen radicals that can attack DNA strands. Of all of these oxidative lesions, 8-oxo-dG lesions are the most prevalent and are predominantly repaired by the human 8-oxoguanine DNA glycosylase-1 (hOGG1) enzyme via the BER pathway, the function of which is dependent on CSB [24].

The bulk of chromatin assembly occurs during the S phase of cell division, when a cell is undergoing replication [5]. The duration of cell cycle arrest also determines the amount of DNA repair occurring during this stage. The duration of S-phase entry delay triggered by G1/S checkpoint is proportionately beneficial to the removal of DNA lesions [25]. Prolonged cell cycle arrest leads to a dramatic change in transcription, as well as post-translational modifications of histone tails, resulting in more efficient lesion removal [26]. The transcription factor and tumour suppressor P53 is known to interact with NER-associated regulatory proteins and thus is important in the NER response. Further, cells which commonly undergo P53 mutations preferentially undergo DNA repair and not apoptosis [27]. Mammalian NER-deficient mutants are also hypersensitive to UV radiation, which is mainly due to induction of apoptosis [28,29], showing that the main apoptosis trigger is due to unrepaired photoproducts. This was further confirmed in experiments with CPD-photolyase transfected cells, showing that the removal of lesions from DNA avoids apoptosis induction [30,31]. Although the formation of CPDs and 6–4PPs is the primary initiator of apoptosis, induction of these lesions is not enough to induce apoptosis [32]. If the lesion is not adequately repaired via NER, it can replicate and form more serious secondary lesions referred to as double-strand breaks (DSBs), forcing the cell into induced death so that they do not undergo cellular proliferation and oncogenic transformation, leading to the development of cancer.

### 2.2. Apoptosis

Apoptosis is an essential process of the cell involved in maintaining homeostatic control of cell number and eliminating cells capable of inflicting harm to an organism. Cells with damaged DNA that is too extensive to be repaired are removed by apoptosis. Thus, a defect in this process contributes to many diseases, including cancer. When a cell is inflicted with damage, this causes the blockage of DNA replication, leading to the collapse of replication forks and lesion formation, inducing apoptosis. This causes the cell to shrink and the chromatin to condense, degrade and separate into individual bodies, a phenomenon known as membrane blebbing [33]. A family of cysteine-dependent aspartate-directed proteases, named caspases, is responsible for the proteolytic cleavage of cellular proteins, internucleosomal DNA fragmentation and propagation of death signalling. This leads to the engulfment of these apoptotic bodies by neighbouring macrophages and dendritic cells [34]. The process of apoptosis is different to other forms of cell death such as necrosis, which is a premature passive process that results in the breakdown of the cell wall in response to environmental stress [35].

Apoptosis functions through two major pathways in mammalian cells, depending on the stimulating factor: the intrinsic and the extrinsic pathways. Both of these pathways require the recruitment of the cell death initiator caspases-8, -9 and -10. Numerous stresses such as DNA-damaging agents and cytotoxic drugs activate the intrinsic pathway, perturbing the structure and function of the mitochondrial outer membrane, releasing several intermembrane proteins, such as cytochrome c. Cytochrome c and APAF-1 (apoptotic protease activating factor-1) form a large oligomeric complex named the apoptosome, leading to the recruitment of the caspase-9 enzyme and cell death. On the other hand, the extrinsic pathway functions through specific cell surface death receptors such as tumor necrosis factor-α (TNF) and Fas. The ligand binding of these receptors leads to recruitment of cytosolic proteins such as the Fas-associated death domain protein (FADD) and caspase-8 and -10, resulting in the formation of the death-inducing signalling complex [36,37].

Apoptosis of a cell can be triggered by many endogenous or exogenous sources. However, in skin cells, one of the most common routes of damage is by exposure to UV. One of the hallmarks of keratinocytes exposed to high dose UV is the induction of apoptosis when high levels of genetic damage are induced [37]. UV-mediated apoptosis is a highly complex process, in which there is a synergistic contribution from many molecular pathways, and thus not all of these can be covered by this review. Exposure of the cell to UV causes the blockage of DNA replication, leading to the collapse of replication forks and DSB formation. If DNA damage is not efficiently repaired, the arrested cells may undergo P53-dependent apoptosis. In response to UV damage signalling, P53 undergoes extensive post-translational modifications at specific residues, resulting in activation of numerous downstream genes including P21, GADD45a and MDM2, leading to activation of caspases-9 and -3 [38,39]. Mice with absent P53 are more prone to UV-induced damage [38] and keratinocytes with knocked down P53 show increased cell death via lower levels of anti-apoptotic proteins [39]. Also, keratinocytes lacking GADD45a display highly reduced apoptotic sunburn cell formation [40]. However, the effect of P53 on apoptosis/repair varies in tissue types [27]. UV-induced apoptosis can also be initiated via the extrinsic pathway by several members of the TNF (tumour necrosis factor) family, including Fas ligand (or CD95L) and TNFR-1 and two tumour necrosis factor-related apoptosis-inducing ligand (TRAIL) receptors, in turn leading to caspase activation [41,42]. Inhibition of caspase-3, the downstream protease of the CD95L signaling pathway, inhibits UV-induced apoptosis, while blockade of caspase-8 only inhibits UV-induced apoptosis partially, showing that UV-mediated DNA damage is not dependent on caspase-8 [37]. Recent studies have revealed that the intrinsic mitochondrial pathway plays an essential role as an integrator and coordinator of UV-induced cell death pathways in keratinocytes. However, the exact contribution of certain caspase-initiated pathways remains to be thoroughly investigated [42].

### 2.3. Structural Changes of Chromatin Following UV

UVB radiation is known to be a potent agent for the initiation of programmed cell death. Although chromatin is known to undergo conformational changes following DNA damage and during apoptosis, the direct evidence of this is limited and more research is required into UV effects on chromatin structure.

In rat glioma cells, UV induces chromosomal giant DNA formation, followed by internucleosomal DNA fragmentation associated with apoptosis via caspase activation and large-scale chromatin structural changes [43]. In permeabilised erythroleukemia cells, varying doses of UV treatment also had differing effects on the chromatin of the cells. In response to low doses of UV radiation (6 J/m^2^), only small structural changes in the chromatin were noted. However, higher doses of UV (24 J/m^2^) manifested in the reversal of permeabilisation of the cell and the blocking of chromatin condensation at its fibillary stage, covering the chromosome with fibillary chromatin. This caused the prevention of the opening of the nucleus, forcing the cells into apoptosis, most likely due to repair factors not being able to access the site of the DSB [44]. However, the amount of apoptotic bodies observed was much less than that of gamma irradiation-induced DNA damage [45]. In UV-treated mouse microglial cells, UV-induced apoptotic cells showed condensed chromatin accumulating at the nuclear periphery. Surprisingly, the chromatin did not undergo fragmentation, but instead translocated to the cytoplasm [46]. This suggests that UV-induced chromatin degradation is not restricted to the nucleus alone, but this has not been shown for other cell models. Lastly, in the human HaCaT keratinocyte cell line, UVB irradiation is found to cause rapid increases in the formation of CPDs and chromatin condensation, leading to increased apoptosis [47].

## 3. The Role of Histone Modifications and Chromatin Remodeling Complexes in Response to UV-Induced DNA Damage

### 3.1. Recognition of a Lesion by γ-H2AX and the Role of Chromatin Remodeling Complexes

**Figure 2:**
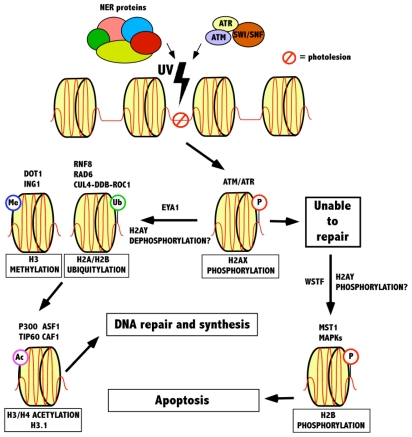
The involvement of histone modifications in cell decisions following UV damage. Chromatin consists of DNA wrapped around nucleosomes in higher order structures. UV damage causes damage to this DNA in the form of photolesions, influencing the recruitment of ATM/ATR, SWI/SNF and an arrangement of NER proteins. ATM/ATR is responsible for the phosphorylation of histone H2AX, serving as a binding site for various repair and checkpoint proteins. This is followed by H2A/H2B ubiquitylation and H3 methylation, as well as acetylation of H3 and H4 histones, leading to repair synthesis. However, if repair is not possible, histone H2B is phosphorylated, influencing apoptosis.

The association of DNA with histones in chromatin prevents DNA repair proteins from accessing photolesions. However, this barrier may be overcome if the DNA lesions change the structural properties of nucleosomes, promoting enzymes to repair these lesions. The cell has several mechanisms by which chromatin can be manipulated to access DNA. These include ATP-dependent chromatin remodeling complexes, incorporation of histone variants into the nucleosome and covalent histone modifications [48]. During interphase, the relaxed chromatin of the eukaryotic cell undergoes a distinct change, resulting in the formation of a highly condensed mitotic chromosome. The biochemical mechanisms responsible for apoptosis, such as chromatin condensation, DNA fragmentation, and release of nuclear proteins, although commonly used as markers for apoptosis, are not fully understood [49]. Post-translational modifications of histone tails such as phosphorylation, acetylation, ubiquitylation and methylation, as well as nucleosome remodelling are also evident in the dynamic changes that occur during the condensation/relaxation process of a cell (Table 1). All of these are important in the response of chromatin to UV-induced DNA damage, including repair and apoptosis (Figure 2).

ATP-dependent chromatin remodeling complexes (CRCs) are divided into 4 families, including the ISWI (imitation of switch), SWI/SNF (switch/sucrose non-fermenting), INO (inositol) and CHD (chromodomain helicase/ATPase DNA binding protein) families. All of these CRCs are important in the chromatin remodelling process, and exhibit mechanistic differences [50]. These CRCs contact both DNA and histones, allowing for nucleosome repositioning or removal. Although they are known to play a role in transcription, recently many of these CRCs have been found to play key roles in the repair of DSBs in yeast, functioning by allowing access of repair proteins and critical histone modifications at the DSB site following DNA damage.

Core histones are mostly globular molecules, although they also possess an unstructured *N*-terminal tail where many post-translational modifications occur [48]. Phosphorylation, ubiquitylation and methylation of proteins plays a primary role in the DNA damage response by facilitating access of various repair proteins to DNA breaks, as well as cell signalling and promoting chromosomal stability. Acetylation plays an important role downstream of these modifications, important for cell cycle progression and the stabilization of chromatin post DNA damage [51]. Upon the formation a of DSB, DNA repair pathways, as well as signals for checkpoint activation to arrest the cell cycle are activated by the cell. The three phosphatidylinositol-3 kinase-like kinases (PIKKs), ataxia-telangiectasia-mutated (ATM), ataxia-telangiectasia-Rad3 related (ATR) and DNA-damage-dependent protein kinase (DNA-PK) are first activated, signaling the presence of a DSB to cell cycle machinery. Although all of these recognize DSBs, ATM and DNA-PK mainly function after ionizing radiation, whereas ATR mainly responds to UV irradiation [52,53]. These in turn phosphorylate P53, either leading to the activation of cell cycle arrest proteins such as P21 and Chk2 or pro-apoptotic proteins such as Bax and various caspases [54].

These three PIKKs are all also responsible for the phosphorylation of the H2A histone variant H2AX (γ-H2AX) at Serine-139 on the *C*-terminal tail, which serves as a binding site for repair and checkpoint proteins, allowing for DNA repair events to be facilitated. Phosphorylated P53 rapidly co-localises and forms a complex with γ-H2AX [55]. It also forms during early apoptosis, induced by death receptor agonists and stimuli that activate the intrinsic apoptotic pathway [56]. This histone variant is found in higher eukaryotes and constitutes approximately 10% of total mammalian H2A. γ-H2AX is initiated in the nuclear periphery adjacent to the nuclear envelope while H2AX remains in the nucleus. This is referred to as the “γ-H2AX ring”. This phosphorylation event is the earliest histone modification event following induction of a DSB and is the most characterized modification following DNA damage [51,52,56]. Caffeine, a known inhibitor of PIKKs, prevents the induction of γ-H2AX and as such increases the amount of cells undergoing UV-induced apoptosis due to a DNA repair defect [57,58]. The mediator of DNA damage checkpoint protein 1 (MDC1) also binds directly to γ-H2AX at sites of DSBs and this is vital in the recruitment of repair factors such as MRE11, RAD50/51, NBS1, FANCD2, 53BP1 and BRCA1 [59,60]. This thereby mediates checkpoint signaling to effector kinases CHK1 and CHK2 [61] and further ATM recruitment results in enhanced accumulation of repair factors.

The chromatin remodeling SWI/SNF complex binds to γ-H2AX, and knockout of the SWI/SNF complex results in inefficient CPD removal and increased DNA sensitivity following DNA damage [62–64]. Therefore, the SWI/SNF complex promotes H2AX phosphorylation by directly acting on chromatin. Recently, Lee and colleagues [65] found that the catalytic ATPase subunit of SWI/SNF, BRG1, binds to γ-H2AX nucleosomes by interacting with acetylated H3 through its bromodomain. When BRG1 was deleted, they found that this caused a significant defect in the repair of DSBs. Moreover, if SWI/SNF or BRG1 were inactivated, the cells became highly susceptible to DNA-damage induced apoptosis due to a prolonged activation of P53 [101]. The alternative ATPase subunit of SWI/SNF, BRM has also been shown to be a tumour suppressor in various tissues. Both BRG1 and BRM were found to be downregulated from the progression of a benign skin lesion into malignant skin cancers, suggesting that these subunits could play an important role in the response to UV-induced DNA damage [102]. Recently, a novel hotspot mutation in a highly conserved region of BRM was discovered in non-melanoma skin cancer, however the effect of this mutation remains elusive [103]. Moreover, cells from BRM knockout mice undergo UV-radiation induced apoptosis more readily than cells from normal mice [104] and this is likely due to cleavage of BRM by cathepsin G [105].

A study with SWI/SNF deficient cells showed that these cells had highly reduced levels of the P53-regulated proteins P21 and GADD45 following UV-induced damage, suggesting that SWI/SNF promotes apoptosis via induction of these two cell cycle control proteins [63]. It should also be noted that SNF5, another subunit of the SWI/SNF complex, has also been found to be important in the DNA damage response. Cells with knockout of SNF5 led to a hypersensitive DNA damage response, and also an aberrant apoptotic effect and change in P53 activity, leading to a proliferative defect [106,107]. Other studies have also shown that SNF5 interacts with UV damage recognition factor XPC and co-localises with XPC at the damage site. SNF5 deficiency led to a defect in downstream ATM and H2AX phosphorylation [108].

The chromatin-remodeling INO80 complex is also recruited to the site of a DSB via interaction with γ-H2AX, where it is thought to induce histone eviction so as to facilitate the access of repair proteins to the site of damage, promoting the restoration of chromatin structure [109,110]. The SWR1 complex, a member of the INO80 subfamily, has also been shown to make a direct contribution to DSB repair via association with the γ-H2AX complex [111]. The chromatin-remodeling protein RSC is also required for DSB repair following synapsis for the completion of the recombinational repair event [62]. The phosphorylation of γ-H2AX also serves as a platform for other complexes. It is required for the recruitment of the TIP60-HAT complex, integral in the DNA damage response in mammals [112]. TIP60 in turn acetylates H4 at multiple sites, required for the recruitment of other DNA damage proteins [113]. It should also be noted that γ-H2AX dephosphorylation by wild-type P53-induced phosphatase (WIP1) is also necessary following recovery from checkpoint arrest and cell-cycle progression following chromatin recovery from UV-induced DNA damage [114]. In repair-deficient XP-B cells, which lack functional XPB DNA repair helicase, γ-H2AX induction still occurs following UV-induced damage, as well as phosphorylation of ATM and NBS1, suggesting that these events still occur in the absence of NER [60]. The activation of P53 was prolonged in these XP-B cells, thus failing to induce downstream targets such as WIP1, GADD45A and MDM2, inhibiting apoptosis [60,115]. Further, overexpression of WIP1 blocks recruitment of the repair proteins MDC1 and 53BP1 to UV-induced damage sites, inhibiting repair and promoting apoptosis [115]. These findings suggest an important role for WIP1 in apoptosis.

Collectively, these data show that ATP-dependent CRCs form a cooperative feedback activation loop with γ-H2AX, influencing the recruitment of repair factors and checkpoint proteins to assist in DSB repair. Moreover, upon inefficient repair of DNA, this dynamic interaction can encourage the apoptotic response of a cell. This interaction is vital in both the repair and apoptotic signalling of a cell following UV-induced DNA damage. However, it is unclear whether these chromatin remodeling pathways directly regulate this response or if it is due to other indirect pathways.

### 3.2. Modifications Following γ-H2AX in the UV-Induced DNA Damage Response

**Figure 3:**
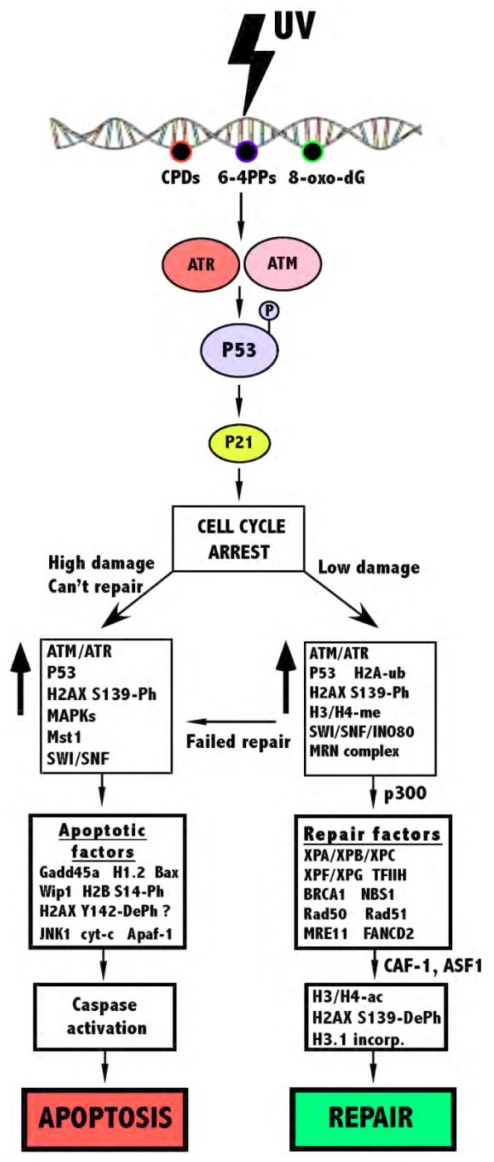
The effect of histones and activated enzymes post UV. P/Ph = phosphorylation, DePh = dephosphorylation, Ac = acetylation, Ub = ubiquitylation, Me = methylation. Upon UV-induced DNA damage, photoproducts are formed, triggering the ATM/ATR response, in turn phosphorylating P53 and activating P21, leading to cell cycle arrest. The amount of damage, as well as histone modifications and protein activation then determines the fate of the cell.

Following phosphorylation of H2AX, many other chromatin events take place, affecting whether a cell recovers from UV-induced DNA damage via NER or is forced into apoptosis (Figure 3). It has been shown that phosphorylation of histone H2A, H2B, H3 and H4, dephosphorylation of histone H1, acetylation of H2B and H4, as well as methylation of histone H3 and H4 are all associated in the apoptotic process [116]. However, the apoptotic response of the cell following UV-irradiation still remains elusive.

Histone H2B phosphorylation at serine-14 (S14) is a hallmark of apoptosis and has been associated with the condensation of chromatin [66,67]. The phosphorylation of H2B at S14 is mediated by mammalian sterile twenty (MST1) kinase, following caspase-3 mediated cleavage of MST1 [67]. Recently, it was also shown that H2BS14 phosphorylation occurred following UVB irradiation and this was mediated by activated MAPKs (such as ERK1/2, JNK1/2 and P38), showing that H2BS14 phosphorylation is mediated by both MAPKs and caspase-3/MST1 pathways [68]. Moreover, SWI/SNF binds to the H2B N-terminus in yeast [69], and mutation or deletion of the H2B *N*-terminal domain leads to an increased UV sensitivity [70]. This is most likely due to an inability to recruit SNF5 leading to a defect in NER due to altered chromatin structure, and thus a high UV sensitivity [71]. Ubiquitylation of histone H2A also occurs in response to UV radiation [95], as well ubiquitylation of H2B [98], H3 and H4 [99] although these latter modifications are to a much lower extent in comparison to H2A. The ubiquitylation of H2A is dependent on RNF8, which is recruited to sites of UV damage, and is essential for the recruitment of repair factors 53BP1 and BRCA1 [96].

A correlation between early apoptotic dephosphorylation of H1 and the onset of chromatin fragmentation has also been described [72]. However, more recent evidence has suggested that the dephosphorylation of H1 may not be a prerequisite to the onset of apoptotic DNA fragmentation [73]. Moreover, histone H1.2 is increased and forms a protein complex with APAF-1, caspase-9 and CYT-C in response to UV irradiation, showing that histone H1.2 acts a positive regulator of apoptosome formation [74]. A reduction in histone H1.2 expression also inhibits DSB induced apoptosis in thymocytes [75]. The phosphorylation of H4 at serine-1 has also been found to be induced by DNA damage and this is mediated by casein kinase 2 (CK2) [76].

Methylations on histone H3 and H4 are also known to occur in response to UV irradiation and are important in gene regulation and chromatin structure. Although most damage related histone modifications occur at the *N*-terminus, many have been identified to occur within the core portion of these proteins. Methylation of lysine-79 in histone H3 (H3K79), a highly conserved modification catalysed by histone methyltransferase DOT1 [79] also occurs following UV-induced DNA damage. Methylated H3K79 serves as a binding site for RAD9/53BP1 that has involvement in several damage checkpoints [80,81]. In yeast *Saccharomyces cerevisiae,* it was found that mutated H3K79 affected the response to UV-induced damage. Furthermore, mutation of lysines in the H4 *N*-terminus altered the distribution of H3K79 methylation states, showing that these modifications coordinate DNA damage checkpoint and repair pathways [82,83]. Impaired NER seen in these mutants is most likely due to increased binding of SIR complexes, as deletion of SIR1 or SIR2 in these mutants significantly increases the amount of CPD removal following UV irradiation in yeast [84].

SNF5-inactivated T-cell lymphomas showed increased EZH2, a Polycomb (PcG) group protein, which is highly expressed in various cancers. EZH2 is known to mediate gene silencing by catalyzing trimethylation of histone H3K27 at the promoters of various target genes such as P16. SNF5 deficiency in these cells led to fully penetrant and rapid tumour formation, largely due to a defect in cellular senescence [117]. Several other PcG proteins are also involved in the methylation and ubiquitylation of histones and are recruited to sites of DNA damage [118]. BMI1 and RING2 are required for the accumulation of ubiquitylated γ-H2AX at DSB sites [97]. These proteins are believed to be recruited in a poly (ADP ribose) polymerase (PARP)1/2 manner [119]. The DNA repair and apoptotic activities of the tumour suppressor ING1 requires interaction with trimethylated histone H3 lysine 4 [85]. Overexpressed ING1 activates P53 target genes, including P21 and proapoptotic Bax, promoting P53-dependent apoptosis [120,121]. Mutations of ING1 decrease H3K4 trimethylation, and these mutants have impaired UV-induced DNA repair and apoptosis [85].

Although the signalling pathways and modifications mentioned above which occur in response to DNA damage are quite well depicted, the precise molecular decisions which determine the strategy of the cell in either repairing/surviving or undergoing cell death are not completely understood, in particular the response to UV. However, one recently discovered modification might serve to answer these questions in the future. The *C*-terminus of H2AX shows a tyrosine (Tyr 142 in mammals) in metazoans, however, this is not present in other organisms such as yeast. This particular site is phosphorylated following DNA damage by WSTF (Williams-Beuren syndrome transcription factor), a component of the ISWI ATP-dependent chromatin-remodeling complex, and this phosphorylation promotes the recruitment of pro-apoptotic factors to γ-H2AXS139, inhibiting the recruitment of repair factors to this site [77,78]. Furthermore, Tyr 142 (Y142) dephosphorylation is also dependent on EYA1/3 phosphatases and deletion of either of these phosphatases results in increased Y142 phosphorylation. An increase in apoptotic cell death was observed when EYA phosphatases were knocked out in response to DNA damage and EYA was found to interact with H2AX in the context of chromatin. EYA is recruited to H2AX foci in response to DSBs and this was found to be dependent on the phosphorylation of both ATR/ATM. Peptides corresponding to the *C*-terminal domain of H2AX with phosphorylation of both S139 and Y142 were constructed and it was found that peptides lacking phosphorylation marks or where Y142 were mutated failed to interact with MDC1, a critical protein for the signaling of downstream repair factors [78]. If repair of the cell is possible, Y142 is dephosphorylated and the DNA damage response machinery is recruited into chromatin in regions flanking the lesion. However if the damage is too severe, Y142 phosphorylation remains and apoptosis is promoted by the association with γ-H2AX and pro-apoptotic JNK1 [78]. It has also been proposed that the γ-H2AX ring together with H2B S14 phosphorylation and H2AX Y142 phosphorylation form a “histone phosphorylation apoptotic code” following formation of a DSB, vital for the repair and apoptotic response of the cell [56,116]. These data suggest that the Y142 site is important for both repair and apoptosis following induction of DSB, however to date this site has not been investigated in response to UV irradiation, but it is possible that it is important for the cellular response since γ-H2AX foci induction is already known to be imperative in the response to UV-induced DNA damage.

### 3.3. Maintenance of Chromatin Following Repair—Histone Acetylation

Recently, the acetylation of core histones on the amino-terminal tails of lysine residues, mediated by histone acetyltransferases (HATs) has been of much clinical interest. This is due to the fact that this acetylation is indeed reversible, catalysed by histone deacetylase (HDAC) enzymes. This deacetylation results in a more compacted chromatin structure, inhibitory to many cellular processes. This in turn affects the formation of UV-induced DNA lesions due to this change in chromatin conformation, preventing transcription and entry of cells into S phase. With the use of histone deacetylase inhibitors such as Trischostatin A (TSA), it is possible to sensitise cancer cells to UV-induced apoptosis, as well as promoting growth arrest and differentation [122,123], thus promoting an anti-cancer effect. Acetylations of histones H3 and H4 have been shown to play a role in the UV damage response. The release of HDACs and the subsequent acetylation of histone H3 and H4 ensures progression from the G_1_ to S phase of the cell cycle and the restructuring of chromatin post DNA damage [87]. These events have been found to follow modifications such as H2AX phosphorylation and H3K79 methylation, and are important in both the opening and restoring of chromatin following DNA damage. Induction of maximum core histone acetylation enhances NER following UV irradiation [16].

The P300 histone acetyltransferase mediates the UV-induced acetylation of H3 histones. Inactivation of P300 prevents UV-induced H3 acetylation as well as activation of γ-H2AX and P53, indicating that P300 is involved in UV induction of γ-H2AX and P53. Furthermore, UV-induced acetylation of histone H3 does not occur in the absence of P53 [88]. RAD16, a catalytic subunit of SWI/SNF, also mediates hyperacetylation of H3 histones in yeast [84]. In heterochromatic regions in *Drosophila* cells, a decrease in trimethylated histone H3 lysine-9 levels is found in response to UV-induced DNA damage and this process is dependent on a fly homolog of P53 [86]. Acetylation of H3K9 also increases following UV irradiation [89] and this process is also dependent on this P53 homolog. This result suggests that crosstalk between these histone modifications may occur following UV irradiation. Upon DNA damage, a histone demethylase may be recruited, reversing the trimethylation of the H3K9 site, permitting acetylation by HATs and an increased NER response [16].

In response to UV irradiation, the histone chaperone anti-silencing function-1 (ASF1) is important for the DNA damage checkpoint by facilitating the acetylation of histone H3 on lysine 56 by the histone acetyltransferase RTT109 [90]. Knockdown of ASF1 post-UV results in defective H3K79 acetylation as well as a fault in γ-H2AX dephosphorylation, and this was regulated by the damage response protein ATM. Thus, ASF1 and the acetylation of H3K79 are integral for UV-induced cell cycle arrest [91]. It should also be noted that UV irradiation also results in increased acetylation levels of H3K14 and H3K18 in *Drosophila*, as well as phosphorylation at serine 10. The loss of P53 perturbs the normal regulation of S10 phosphorylation, as well as H3K14 but not H3K18 acetylation, affecting recovery of cells from G_2_-M arrest, most likely forcing cells into apoptosis [92]. The acetylation status of histone H4 is also dramatically decreased in UV irradiated G_1_ phase checkpoint cells, suggesting that acetylation of core histones is necessary for the G_1_/S transition [93]. Both hypoacetylated and trimethylated histone H4 is also released from nuclei early in apoptosis [94].

If DNA is successfully repaired, a variant of histone H3, H3.1, becomes incorporated at sites of UV-induced DNA damage *in vivo,* and this event is facilitated by a histone chaperone named chromatin assembly factor 1 (CAF-1) outside of S-phase, contributing to the maintenance of chromatin assembly [100]. These observations show that new histones can also become incorporated that do not contain the modifications present in histones before the DNA was damaged.

## 4. Conclusions

The maintenance of genomic stability following UV-induced DNA damage in eukaryotic cells is a complex process, in which histone modifications and nucleosome remodelling via ATP-dependent chromatin remodelling complexes, such as SWI/SNF, are tightly regulated. Cross-talk between these histones is critical in the dynamic response of chromatin to UV damage to signal and recruit repair factors and pro-apoptotic proteins. However, much more research must be done to understand these interactions. The discovery of novel modifications such as H2AX Y142 and its interaction with other histone modifications, chromatin remodelling complexes and damage response enzymes such as P53 are likely to shine more light on the response of a cell to UV as more is discovered about this site and its various interactions. It will be important to determine whether this site is indeed important in the UV-induced DNA damage response. A greater understanding of the many changes following histone modifications to UV-induced damage could undoubtedly lead to an improved outcome for patients. This is already apparent in drugs being trialled such as the histone deacetylase inhibitor TSA, which is showing great promise in reducing UV-induced cellular transformation in humans.

## Figures and Tables

**Table 1:** Post-translational modifications following UV damage and their association with repair and/or apoptosis. ND = Not determined.

Histone residue	Modification type	Enzymes involved	Associated with?	References
**H2AX S139**	Phosphorylation	ATM/ATR, DNA-PK, P53, MDC1, SWI/SNF	Repair and apoptosis	[50–65]
**H2B S14**	Phosphorylation	MST1, MAPKs	Apoptosis	[66–71]
**H1** **H1.2**	Phosphorylation/Dephosphorylation	ND	Apoptosis	[72–76]
**H2AX Y142 (?)**	Phosphorylation	WSTF	Apoptosis	[77]
**H2AX Y142 (?)**	Dephosphorylation	EYA1	Repair	[78]
**H3 K79** **H3 K4** **H3 K9**	Methylation	DOT1, SIR ING1 P53	Repair and apoptosis	[79–84] [85] [16,86]
**H3**	Acetylation	P53, P300, ASF1, TIP60	Repair	[87–92]
**H4** **H2A**	Acetylation	ND RNF8, PcGs	Repair	[93,94] [95–97]
**H2B** **H3/H4**	Ubiquitylation	RAD6 CUL4-DDB-ROC1	Repair	[98] [99]
**H3.1**	Incorporation	CAF-1	Repair	[100]

## Abbreviations

6-4PP: pyrimidine 6-4 pyrimidone photoproduct
8-oxo-dG: 8-oxodeoxyguanine
γ-H2AX: phosphorylated histone H2AX
APAF-1: apoptotic protease activating factor-1
ASF1: anti-silencing function-1
ATM: ataxia-telangiectasia-mutated
ATP: adenosine 5′-triphosphate
ATR: ataxia-telangiectasia Rad3-related
BER: base excision repair
CAF1: chromatin assembly factor-1
CK2: casein kinase 2
CPD: cyclobutane pyrimidine dimer
CSB: Cockayne syndrome B
CYT-C: cytochrome-c
DNA-PK: DNA-damage-dependent protein kinase
DSB: double strand break
FADD: Fas-associated death domain protein
GADD45a: growth arrest and DNA-damage-inducible alpha
GGR: global genome repair
HAT: histone acetyltransferase
HDAC: histone deacetylase
hOGG1: human 8-oxoguanine DNA glycosylase-1
INO: inositol
ISWI: imitation of switch
MDC1: mediator of DNA-damage checkpoint 1
MDM2: P53 binding protein homolog
MST1: mammalian sterile twenty kinase-1
NER: nucleotide excision repair
PARP: poly(ADP ribose) polymerase
PcG: Polycomb
PCNA: proliferating cell nuclear antigen
PIKK: phosphatidylinositol-3 kinase-like kinase
RF: replication factor
RP: replication protein
SWI/SNF: switch/sucrose
TCR: transcription coupled repair
TFIIH: transcription factor IIH
TNF: tumour necrosis factor
TRAIL: tumour necrosis factorrelated apoptosis-inducing ligand
TSA: Trischostatin A
UV: ultraviolet
WIP1: wild-type P53-induced phosphatase-1
WSTF: Williams-Beuren syndrome transcription factor
XP: Xeroderma pigmentosum complex.
